# Emerging *Leishmania donovani* Lineages Associated with Cutaneous Leishmaniasis, Himachal Pradesh, India, 2023

**DOI:** 10.3201/eid3009.231595

**Published:** 2024-09

**Authors:** Patrick Lypaczewski, Yogesh Chauhan, Kayla Paulini, Lovlesh Thakur, Shailja Chauhan, Ezrah Isaac Roy, Greg Matlashewski, Manju Jain

**Affiliations:** McGill University, Montreal, Quebec, Canada (P. Lypaczewski, K. Paulini, E.I. Roy, G. Matlashewski);; Central University of Punjab, Bathinda, India (Y. Chauhan, M. Jain);; Cleveland State University Center for Gene Regulation in Health and Disease, Cleveland, Ohio, USA (L. Thakur);; Indira Gandhi Medical College Cancer Hospital, Shimla, India (S. Chauhan)

**Keywords:** leishmaniasis, Leishmania donovani, cutaneous leishmaniasis, hybrid parasites, whole-genome sequencing, Himachal Pradesh, surveillance, parasites, India

## Abstract

The clinical manifestation of leishmaniasis has historically been determined by the *Leishmania* species involved. However, recent emergence of novel *Leishmania* lineages has caused atypical pathologies. We isolated and characterized 2 new *Leishmania donovani* parasites causing cutaneous leishmaniasis in Himachal Pradesh, India.

Leishmaniasis is a neglected tropical disease caused by the protozoan parasite *Leishmania*. The manifestation of the disease has historically been species-specific: *Leishmania donovani* and *Leishmania infantum* cause visceral leishmaniasis (VL), also called kala-azar, and many species such as *Leishmania tropica* and *Leishmania major* cause cutaneous leishmaniasis (CL) (*1*). In recent years, however, the existence of interspecies and intraspecies hybrids has emerged, and hybridization has been associated with a potential cause of CL in Sri Lanka (*2*) and Himachal Pradesh, India (*3*). In Sri Lanka, CL is mostly caused by an atypical *L. donovani* (*4*–*6*) and CL cases were recently observed to be associated with *L. donovani*/*L. major* hybrids or *L. donovani*/*L. tropica* hybrids (*2*). CL is an emerging disease in Himachal Pradesh, where a recently identified *L. donovani* intraspecies hybrid isolated from a CL patient belonged to the Indian subcontinent 1 (ISC1) Yeti clade (*3*). Further, the recent discovery of ISC1 *Leishmania* parasites in the neighboring region of West-Nepal supports the establishment of the ISC1 clade in the area (*7*). Therefore, continued monitoring for emergence of CL in Himachal Pradesh is necessary to identify new *L. donovani* lineages associated with cutaneous disease outcomes. We report 2 new cases of CL in Himachal Pradesh caused by *L. donovani* belonging to the ISC1 Yeti clade that are not hybrid parasites previously identified from this region (GenBank BioProject no. PRJNA701770) (Appendix Figure 1) (*3*). Genome surveillance of CL parasites coming from Himachal Pradesh can help identify gene sequences associated with CL disease outcomes and identify the origin and transmission of emerging *L. donovani* parasites.

We performed a phylogenetic analysis by adding LdHPCL71 and LdHPCL76 to a previously generated tree containing 685 whole-genome *L. donovani* isolates (*3*). As shown previously, the topology of the tree matches the geographic origin of the samples used (*2*). Consistent with the previous report from Himachal Pradesh (*3*), phylogenetic analysis of the new LdHPCL71 and LdHPCL76 CL lineages revealed that they also clustered within the ISC1 Yeti clade of *L. donovani* (Figure). Because the previously identified cutaneous lineage LdHPCL66 from Himachal Pradesh was an intraspecific hybrid (*3*), we next investigated whether the LdHPCL71 and LdHPCL76 parasites were also hybrids. We used VarScan (https://varscan.sourceforge.net) to identify all single-nucleotide polymorphisms (SNPs) by using the Sri Lanka CL *L. donovani* reference strain (*6*) and compared the SNP frequencies with previous data on hybrid parasites (Appendix Figure 2, panel A). We plotted the full genomic representation for all 36 chromosomes for each parasite by using Circos (https://circos.ca) (Appendix Figure 2, panel B). 

**Figure:**
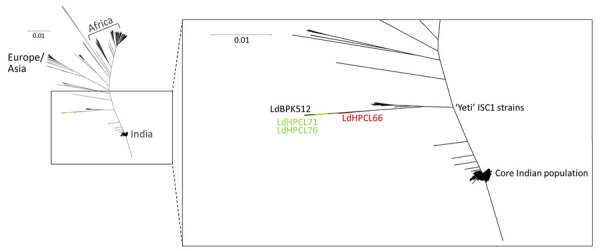
Phylogeny of *Leishmania donovani* lineages associated with cutaneous leishmaniasis, Himachal Pradesh, India, 2023. Novel *L. donovani* from this study (green) are compared with the global population of the 684 parasites previously reported in the *L. donovani* complex, including reference strains *L. donovani* LV9, *L. donovani* BHU 1220, and *L. donovani* BPK282A1. The previously isolated interspecific hybrid LdHPCL66 (red) falls halfway between the unique LDBPK512 and Yeti-ISC1 lineages. The novel LdHPCL71 and LdHPCL76 (green) nonhybrid parasites are more closely related to the unique LdBPK512 parasite. Scale bar indicates the modified Euclidian distance as calculated by TASSEL (https://tassel.bitbucket.io).

The heterozygosity index of LdHPCL71 was 0.168 and of LdHPCL76 was 0.158, suggesting that they are not hybrids because the known hybrids have been shown to contain a large portion of heterozygous SNPs (*2*,*3*). In addition, the many SNPs seen (Appendix Figure 2, panel B) are homozygous, indicating the lineages are distant from the core *L. donovani* parasite population in India but neither parasite seems to be a hybrid. We compared the SNPs from each of the newly identified parasites with the SNPs from the previously isolated LdHPCL66 hybrids from the Yeti ISC1 group to determine if a subset of SNPs is common among the 3 lineages (Appendix Table). We found too many SNPs in common between the parasites to specifically associate any of them with the CL manifestations in patients because they are from 3 divergent lineages in an undersampled population.

Our data, combined with other reports of ISC1 spread (*7*), could support the theory that atypical *L. donovani* parasites are being increasingly encountered as a cause of CL in the Indian subcontinent. That hypothesis is further supported by a recent report on occurrence of CL cases in provinces in Nepal caused by *L. donovani* that are endemic and nonendemic for visceral disease (*8*). Of potential concern, this clade now includes both hybrid and nonhybrid parasites able to cause VL and CL. Emergence of such CL-causing *L. donovani* parasites highlights the urgent need for molecular surveillance as an integral part of the ongoing kala-azar elimination program in the Indian subcontinent. The ongoing regional strategic framework emphasizes the need to control post–kala-azar dermal leishmaniasis cases as a parasite reservoir to break the transmission cycle for sustaining the elimination program (*9*). On a similar note, CL cases caused by atypical *L. donovani* genotypes may also contribute to the transmission cycle of VL in some patients. Our observations support the argument that surveillance of atypical *L. donovani* lineages associated with CL should be included in VL elimination programs.

Appendix Additional information on emerging *Leishmania donovani* lineages associated with cutaneous leishmaniasis, Himachal Pradesh, India, 2023.
