# Supplementary material for: Emerging Leishmania donovani Lineages Associated with Cutaneous Leishmaniasis, Himachal Pradesh, India, 2023
Source: Emerg Infect Dis. 2024 Sep;30(9):1957–9. doi: 10.3201/eid3009.231595 (PMC11346985; doi:10.3201/eid3009.231595)
Supplement: Appendix — Additional information on emerging Leishmania donovani lineages associated with cutaneous leishmaniasis, Himachal Pradesh, India, 2023. [file 23-1595-Techapp-s1.pdf]

Article DOI: https://doi.org/10.3201/eid3009.231595

Emerging *Leishmania donovani* Lineages Associated with Cutaneous Leishmaniasis, Himachal Pradesh, India, 2023

Appendix

Methods

LdHPCL71 and LdHPCL76 were isolated from CL patients belonging to Kinnaur (6050 m above sea level) and Rampur (1020 m above sea level) regions of Himachal Pradesh (figure s1) with a period of illness from 2.5 to 3 months respectively after the initial appearance of cutaneous manifestation and with no report of travel outside of the state around the disease occurrence time. Patient with LdHPCL71 was a 50-year-old female with a single erythematous, nodular plaque lesion while patient with LdHPCL76 was a 12-year-old girl with a single erythematous elevated popular lesion at the time of diagnosis and sample collection. Informed consent was obtained from the patients with due approval of the study by the Institutional Ethics Committee IGMC, Shimla, H.P., Approval no. HFW (MS) G-5 (Ethics)/2014-10886. Based on sequence analysis of ITS1 and 6 PGDH amplified product, the parasites were identified as *L. donovani.*

Lesional tissue biopsies were processed and parasites were cultured as previously described (*1*) and DNA was extracted from cultures using the standard phenol-chloroform method. DNA was sequenced using an Illumina Hi-Seq instrument in 150bp paired end mode, resulting in an average coverage of 59.6, a mean base quality of 35.8, a mean mapping quality of 53 and a breadth of coverage of 99.56 for LdHPCL71. Likewise, an average coverage of 147, a mean base quality of 35.9, a mean mapping quality of 54 and a breadth of coverage of 99.63 for LdHPCL76.

Whole-genome analysis was performed as previously described (*1*,*2*). Galaxy's bedtools was used to remove SNPs that are common in the ISC region using previously sequenced 'Yeti' parasites (Genbank accession numbers ERR206282 and ERR206270) and to identify SNPs that

were in common between LdHPCL71, LdHPCL76 and the previously identified LdHPCL66 parasites (*1*) (Genbank accession number: SRR13707160) (Appendix Table). Phylogeny analysis was performed as previously described (*2*,*3*). Briefly, the SNPs for all samples were combined and used to generate a neighbor-joining tree using the TASSEL software in Newick format. ItoL was then used to display this tree.

References

1. Lypaczewski P, Thakur L, Jain A, Kumari S, Paulini K, Matlashewski G, et al. An intraspecies *Leishmania donovani* hybrid from the Indian subcontinent is associated with an atypical phenotype of cutaneous disease. iScience. 2022;25:103802. PubMed https://doi.org/10.1016/j.isci.2022.103802

2. Paulini K, Lypaczewski P, Zhang WW, Perera DJ, Ndao M, Matlashewski G. Investigating the *Leishmania donovani sacp* gene and its role in macrophage infection and survival in mice. Trop Med Infect Dis. 2022;7:384. PubMed https://doi.org/10.3390/tropicalmed7110384

3. Lypaczewski P, Matlashewski G. *Leishmania donovani* hybridisation and introgression in nature: a comparative genomic investigation. Lancet Microbe. 2021;2:e250–8. PubMed https://doi.org/10.1016/S2666-5247(21)00028-8

Appendix Table. Common SNPs between LdHPCL71, LdHPCL76 and LdHPCL66*

Chrm	Gene ID	Product Description	SNP (gene)	SNP (protein)
1	LdCL_010010600	hypothetical protein	172A>G	Asn58Asp
	LdCL_010012500	Meckel syndrome type 6 protein, putative	604A>C	Thr202Pro
2	LdCL_020008400	glycosyl transferase, putative	1519C>T	Arg507Trp
	LdCL_020009200	Mitochondrial protein 81	574C>A	Pro192Thr
	LdCL_020009900	hypothetical protein	1387A>C	Ser463Arg
	LdCL_020012500	dehydrogenase/oxidoreductase-like protein	103A>G	Asn35Asp
3	LdCL_030008400	hypothetical protein	2329A>G	Thr777Ala
	LdCL_030010000	hypothetical protein	7346G>A	Arg2449Gln
	LdCL_030010600	quinone oxidoreductase, putative	593T>C	Val198Ala
	LdCL_030012800	serine/threonine-protein kinase, putative	6776C>T	Pro2259Leu
			2000T>C	Val667Ala
4	LdCL_040008000	Tetratricopeptide repeat, putative	799T>C	Phe267Leu
	LdCL_040011100	DENN (AEX-3) domain containing protein, putative	3655G>A	Asp1219Asn
	LdCL_040012000	Zinc finger, C3HC4 type (RING finger) containing protein, putative	137A>G	Asn46Ser
5	LdCL_050012200	CYC2-like cyclin, putative	1664C>G	Ala555Gly
	LdCL_050014700	metallo-peptidase, Clan M-, Family M49	798G>T	Gln266His
	LdCL_050016900	hypothetical protein	127C>A	Leu43Ile
6	LdCL_060007900	hypothetical protein	1220A>G	Gln407Arg
	LdCL_060010700	hypothetical protein	508A>G	Thr170Ala
	LdCL_060014400	Protein phosphatase 2C, putative	3422A>G	Asp1141Gly
	LdCL_060014800	TLD, putative	986A>G	His329Arg
7	LdCL_070007300	hypothetical protein	566C>T	Thr189Ile
	LdCL_070010300	Dynein heavy chain, N-terminal region 2, putative	11969A>G	His3990Arg
	LdCL_070013700	hypothetical protein	604G>T	Val202Leu
	LdCL_070014600	hypothetical protein	5707G>T	Val1903Leu

Chrm	Gene ID	Product Description	SNP (gene)	SNP (protein)
			5767A>G	Met1923Val
	LdCL_070015000	protein kinase, putative	1961A>T	Glu654Val
	LdCL_070015700	protein of unknown function (DUF3437), putative	562G>C	Val188Leu
	LdCL_070015800	hypothetical protein	2369G>A	Gly790Asp
	LdCL_070016500	DNA repair and recombination helicase protein PIF7, putative	2837C>T	Pro946Leu
	LdCL_070018000	proteasome regulatory non-ATP-ase subunit, putative	866C>T	Ala289Val
8	LdCL_080008400	hypothetical protein	9420G>A	Met3140Ile
	LdCL_080008800	hypothetical protein	1376A>C	Gln459Pro
	LdCL_080008900	hypothetical protein	284G>T	Gly95Val
	LdCL_080010200	hypothetical protein	1864C>T	Arg622Cys
	LdCL_080017100	hypothetical protein	169C>T	Pro57Ser
	LdCL_080017800	hypothetical protein	1297C>T	Pro433Ser
	LdCL_080018500	hypothetical protein	1297C>T	Pro433Ser
9	LdCL_090009900	Plus-3 domain/Zinc finger, C3HC4 type (RING finger) containing protein, putative	408A>C	Lys136Asn
	LdCL_090010200	hypothetical protein	2014G>A	Gly672Ser
	LdCL_090011800	hypothetical protein	727G>A	Ala243Thr
			1594C>T	Arg532Cys
	LdCL_090015900	hypothetical protein	916A>G	Thr306Ala
	LdCL_090018800	hypothetical protein	2198G>A	Gly733Asp
	LdCL_090018900	hypothetical protein	130A>G	Met44Val
	LdCL_090019000	hypothetical protein	2744C>T	Ser915Phe
	LdCL_090021500	ABC transporter, putative	55A>G	Ile19Val
10	LdCL_100017300	hypothetical protein	2204C>T	Ala735Val
	LdCL_100018100	protein of unknown function (DUF1042), putative	1786A>T	Ile596Leu
	LdCL_100018600	hypothetical protein	994G>A	Gly332Arg
11	LdCL_110005200	Bacterial transferase hexapeptide (six repeats), putative	280G>A	Val94Ile
	LdCL_110012800	hypothetical protein	1558G>A	Val520Ile
			2308G>T	Ala770Ser
	LdCL_110013500	hypothetical protein	1046C>T	Ala349Val
12	LdCL_120006300	hypothetical protein	1744C>T	His582Tyr
	LdCL_120006600	leucine-rich repeat protein, putative	2840C>T	Ala947Val
	LdCL_120006700	dolichyl-P-Man:GDP-Man5GlcNAc2-PP-dolichyl alpha-1,2-mannosyltransferase, putative	821G>A	Arg274Gln
	LdCL_120006900	hypothetical protein	1945G>A	Glu649Lys
	LdCL_120008000	hypothetical protein	2207G>A	Arg736Gln
	LdCL_120009800	hypothetical protein	40C>T	Pro14Ser
	LdCL_120009900	hypothetical protein	2128T>C	Ter710Arg
	LdCL_120011100	Fusaric acid resistance protein-like, putative	475C>T	Leu159Phe
			5183A>G	His1728Arg
	LdCL_120012600	surface antigen protein 2, putative	1069G>C	Gly357Arg
	LdCL_120012900	hypothetical protein	1835T>C	Val612Ala
	LdCL_120013300	hypothetical protein	1802T>C	Val601Ala
	LdCL_120013800	hypothetical protein	1802T>C	Val601Ala
	LdCL_120014300	hypothetical protein	1802T>C	Val601Ala
	LdCL_120014800	hypothetical protein	1802T>C	Val601Ala
	LdCL_120015300	hypothetical protein	1802T>C	Val601Ala
	LdCL_120015500	surface antigen protein 2, putative	681G>T	Arg227Ser
	LdCL_120016300	hypothetical protein	1802T>C	Val601Ala
	LdCL_120016800	hypothetical protein	1802T>C	Val601Ala
	LdCL_120017300	hypothetical protein	1802T>C	Val601Ala
	LdCL_120017900	surface antigen protein 2, putative	1934A>T	Tyr645Phe
	LdCL_120018500	hypothetical protein	340A>G	Lys114Glu
13	LdCL_130009300	ATP-dependent RNA helicase, putative	418A>G	Ile140Val
	LdCL_130012700	hypothetical protein	1049T>C	Val350Ala
	LdCL_130012800	hypothetical protein	2479C>G	Gln827Glu
	LdCL_130015900	calmodulin, putative	380G>A	Arg127His
	LdCL_130016200	adenylosuccinate synthetase, putative	347C>T	Thr116Met
	LdCL_130018600	hypothetical protein	755T>C	Val252Ala
	LdCL_130019600	PIN domain containing protein, putative	1758C>G	His586Gln
	LdCL_130019900	ATPase family associated with various cellular activities (AAA), putative	886G>T	Ala296Ser
14	LdCL_140007200	hypothetical protein	283G>A	Gly95Ser
	LdCL_140007300	hypothetical protein	319A>C	Ser107Arg
	LdCL_140008700	hypothetical protein	3825C>A	Ser1275Arg
	LdCL_140011500	hypothetical protein	2983G>A	Glu995Lys

Chrm	Gene ID	Product Description	SNP (gene)	SNP (protein)
			469C>A	Arg157Ser
	LdCL_140013600	hypothetical protein	1786G>A	Gly596Arg
	LdCL_140019600	hypothetical protein	1078G>A	Ala360Thr
	LdCL_140020000	TPR repeat/DnaJ domain containing protein, putative	1705G>C	Glu569Gln
15	LdCL_150014600	hypothetical protein	1460A>G	His487Arg
	LdCL_150015900	DNA repair protein Rad9, putative	1662C>G	His554Gln
	LdCL_150018800	actin-like protein, putative	529G>A	Val177Ile
16	LdCL_160005100	hypothetical protein	1022A>G	His341Arg
	LdCL_160019700	EMG1/NEP1 methyltransferase, putative	688T>C	Phe230Leu
	LdCL_160022500	Clustered mitochondria/Translation initiation factor eIF3 subunit 135, putative	2008G>C	Val670Leu
	LdCL_160022600	Flagellar Member 3	2098G>T	Ala700Ser
17	LdCL_170009100	hypothetical protein	398G>A	Arg133Gln
	LdCL_170014000	hypothetical protein	874G>A	Ala292Thr
	LdCL_170014300	hypothetical protein	508A>G	Thr170Ala
	LdCL_170016200	ATP-dependent RNA helicase, putative	2552C>T	Ser851Leu
	LdCL_170018400	NOL1/NOP2/sun family, putative	1505A>G	His502Arg
18	LdCL_180005300	major facilitator superfamily protein (MFS), putative	463T>G	Phe155Val
19	LdCL_190016500	hypothetical protein	3357G>T	Gln1119His
	LdCL_190017400	hypothetical protein	2917C>T	His973Tyr
20	LdCL_200005900	Ion channel/Calcium-activated BK potassium channel alpha subunit, putative	1879A>G	Thr627Ala
	LdCL_200008700	Sugar (and other) transporter/Major Facilitator Superfamily, putative	10A>G	Thr4Ala
	LdCL_200009500	hypothetical protein	3074G>A	Arg1025Lys
	LdCL_200009800	AIG2-like family, putative	1015G>A	Gly339Ser
	LdCL_200011100	hypothetical protein	1285C>T	Arg429Cys
	LdCL_200012500	protein kinase, putative	9772G>A	Glu3258Lys
	LdCL_200012700	tubulin-tyrosine ligase, putative	523C>G	Gln175Glu
	LdCL_200015200	hypothetical protein	904A>G	Thr302Ala
21	LdCL_210011900	phosphoglucomutase, putative	1760T>C	Val587Ala
	LdCL_210024400	NIMA-related kinase, putative	1562A>G	Lys521Arg
	LdCL_210027900	hypothetical protein	1349C>T	Pro450Leu
22	LdCL_220011900	3'a2rel-related protein	1121C>A	Pro374Gln
	LdCL_220013400	Translation initiation factor SUI1, putative	1888A>G	Ile630Val
	LdCL_220014100	3'a2rel-related protein	1121C>A	Pro374Gln
	LdCL_220014200	Protein of unknown function (DUF3184), putative	121C>G	His41Asp
	LdCL_220014400	hypothetical protein	2118A>G	Ter706Trp
	LdCL_220015900	Galactose oxidase, central domain/Kelch motif containing protein, putative	3497T>C	Leu1166Pro
	LdCL_220016600	Zinc finger, C3HC4 type (RING finger) containing protein, putative	6971T>C	Val2324Ala
	LdCL_220018800	hypothetical protein	4750A>G	Thr1584Ala
	LdCL_220019500	hypothetical protein	193A>G	Asn65Asp
	LdCL_220021600	exosome complex exonuclease RRP45-like, putative	300C>A	Asp100Glu
23	LdCL_230006200	Concanavalin A-like lectin/glucanases superfamily/Beige/BEACH domain containing protein, putative	3221A>G	Gln1074Arg
24	LdCL_240007800	hypothetical protein	2831C>T	Thr944Met
	LdCL_240008700	ppg3-related protein-like protein	2698T>C	Ser900Pro
	LdCL_240012100	hypothetical protein	1888A>G	Ile630Val
	LdCL_240014500	hypothetical protein	2411C>T	Ala804Val
	LdCL_240019900	kinesin, putative	2782G>A	Gly928Arg
	LdCL_240020300	ABC transporter transmembrane region/ABC transporter, putative	6566C>T	Thr2189Met
	LdCL_240028200	Protein of unknown function N-terminal domain (DUF2450)/Protein of unknown function C-terminus (DUF2451), putative	1534G>C	Val512Leu
25	LdCL_250006000	Qc-SNARE protein, putative	113G>A	Arg38His
	LdCL_250009000	hypothetical protein	262G>C	Ala88Pro
	LdCL_250010000	Myosin head (motor domain), putative	3910C>A	Pro1304Thr
			152A>G	Tyr51Cys
	LdCL_250013600	DNL zinc finger containing protein, putative	338C>A	Ser113Tyr
	LdCL_250014100	hypothetical protein	553A>C	Ser185Arg
	LdCL_250014200	hypothetical protein	2106G>T	Met702Ile
	LdCL_250015000	hypothetical protein	1668G>T	Met556Ile

Chrm	Gene ID	Product Description	SNP (gene)	SNP (protein)
	LdCL_250015400	dynein heavy chain, putative	7066A>G	Met2356Val
			7840G>A	Val2614Ile
	LdCL_250015600	hypothetical protein	142G>T	Val48Phe
	LdCL_250019700	TAP42-like family, putative	496G>A	Glu166Lys
	LdCL_250023700	hypothetical protein	242G>T	Arg81Met
	LdCL_250029800	Pex2 / Pex12 amino terminal region/Zinc finger, C3HC4 type (RING finger)/Ring finger domain/zinc-RING finger domain/Anaphase-promoting complex subunit 11 RING-H2 finger containing protein, putative	170C>T	Thr57Ile
26	LdCL_260009000	hypothetical protein	5398T>G	Ser1800Ala
	LdCL_260013400	type II (glutathione peroxidase-like) tryparedoxin peroxidase	494T>C	Phe165Ser
	LdCL_260015600	dynein heavy chain, putative	7304A>C	Asp2435Ala
			8242G>A	Val2748Met
	LdCL_260015800	hypothetical protein	191C>A	Thr64Lys
	LdCL_260016100	hypothetical protein	1412A>G	Asn471Ser
	LdCL_260016700	hypothetical protein	1939A>G	Thr647Ala
	LdCL_260017300	hypothetical protein	3560A>G	Gln1187Arg
			5165T>G	Phe1722Cys
	LdCL_260020500	hypothetical protein	95G>C	Arg32Pro
	LdCL_260028500	hypothetical protein	490C>G	His164Asp
	LdCL_260030900	hypothetical protein	1380G>A	Met460Ile
27	LdCL_270009900	calpain-like cysteine peptidase, putative	11552C>T	Pro3851Leu
	LdCL_270010000	calpain-like cysteine peptidase, putative	437G>C	Gly146Ala
	LdCL_270010100	radial spoke protein 3, putative	1033C>T	Arg345Cys
	LdCL_270021400	diacylglycerol acyltransferase, putative	2506G>C	Val836Leu
	LdCL_270022400	BRO1-like domain/ALIX V-shaped domain binding to HIV, putative	681G>A	Met227Ile
	LdCL_270022700	molybdopterin synthase sulfurylase-like protein, putative	71A>C	Lys24Thr
	LdCL_270023700	Frag1/DRAM/Sfk1 family, putative	2765C>T	Ala922Val
	LdCL_270024100	protein kinase-like protein	125T>C	Leu42Ser
	LdCL_270024600	Dual specificity phosphatase, catalytic domain containing protein, putative	4714G>C	Ala1572Pro
	LdCL_270024800	hypothetical protein	2288A>G	His763Arg
	LdCL_270026700	branched-chain amino acid aminotransferase, putative	556G>A	Ala186Thr
	LdCL_270026800	branched-chain amino acid aminotransferase, putative	556G>A	Ala186Thr
	LdCL_270029500	acyl-CoA oxidase, putative	1711C>T	Pro571Ser
	LdCL_270033000	dynein heavy chain, putative	7237G>A	Asp2413Asn
28	LdCL_280013000	RNA polymerase I	885C>A	Ser295Arg
	LdCL_280017300	Leucine Rich repeat, putative	961A>C	Ile321Leu
	LdCL_280020000	DNA polymerase kappa, putative	542C>G	Ala181Gly
	LdCL_280030700	WW domain containing protein, putative	3938C>G	Ser1313Cys
	LdCL_280031400	Putative serine esterase (DUF676), putative	440G>T	Gly147Val
29	LdCL_290008200	Putative GTPase activating protein for Arf, putative	221G>A	Arg74His
	LdCL_290010100	hypothetical protein	13G>A	Ala5Thr
	LdCL_290010300	Exonuclease, putative	886C>T	Pro296Ser
	LdCL_290010400	Exonuclease, putative	1024T>C	Ser342Pro
	LdCL_290021200	hypothetical protein	691A>C	Ser231Arg
	LdCL_290024700	diphthamide synthesis protein, putative	929A>C	Asp310Ala
	LdCL_290026400	hypothetical protein	828G>C	Gln276His
	LdCL_290029100	hypothetical protein	1216C>T	Pro406Ser
30	LdCL_300019900	ama1 protein, putative	116A>C	Asn39Thr
			128G>A	Ser43Asn
			135C>G	Asp45Glu
	LdCL_300020800	Rad17 cell cycle checkpoint protein, putative	2584G>A	Val862Met
	LdCL_300029500	hypothetical protein	9379A>G	Asn3127Asp
	LdCL_300030200	heat shock 70-related protein 1, mitochondrial precursor, putative	1410A>C	Lys470Asn
			1408A>G	Lys470Glu
			1405T>G	Ser469Ala
			1399G>A	Val467Ile
	LdCL_300031900	hypothetical protein	1730A>G	Lys577Arg
	LdCL_300036400	eukaryotic translation initiation factor 3 subunit 7-like protein	315G>T	Lys105Asn
	LdCL_300040500	DNA ligase I, putative	103G>C	Asp35His

Chrm	Gene ID	Product Description	SNP (gene)	SNP (protein)
	LdCL_300042600	V-type ATPase, C subunit, putative	26C>A	Pro9Gln
31	LdCL_310008300	CRAL/TRIO domain containing protein, putative	2320T>G	Ser774Ala
	LdCL_310011200	hypothetical protein	1723T>G	Ser575Ala
			1151C>T	Ala384Val
	LdCL_310019000	Protein of unknown function (DUF3638)/Protein of unknown function (DUF3645)/Zn-finger in Ran binding protein and others, putative	1646A>C	Lys549Thr
	LdCL_310021600	hypothetical protein	154G>A	Ala52Thr
	LdCL_310025200	hypothetical protein	389G>T	Gly130Val
	LdCL_310027600	transcription like protein nupm1, putative	1384G>A	Gly462Arg
	LdCL_310040700	hypothetical protein	437C>T	Ala146Val
	LdCL_310041200	hypothetical protein	2338G>A	Glu780Lys
32	LdCL_320018700	PTHB1 N-terminus/PTHB1 C-terminus, putative	2750C>T	Ser917Phe
	LdCL_320025500	hypothetical protein	1693G>A	Val565Met
	LdCL_320031100	hypothetical protein	2783C>T	Pro928Leu
	LdCL_320031300	NOL1/NOP2/sun family, putative	674C>T	Pro225Leu
	LdCL_320034300	hypothetical protein	535G>A	Ala179Thr
	LdCL_320034800	Guanine nucleotide exchange factor in Golgi transport N-terminal/Sec7 domain containing protein, putative	2248A>G	Thr750Ala
	LdCL_320038900	hypothetical protein	4030C>T	Arg1344Cys
	LdCL_320042200	Uncharacterized protein family UPF0564, putative	191C>T	Pro64Leu
33	LdCL_330009400	hypothetical protein	903G>T	Lys301Asn
	LdCL_330010500	hypothetical protein	1412G>T	Gly471Val
	LdCL_330020600	QA-SNARE protein putative	26C>T	Ser9Phe
	LdCL_330031000	hypothetical protein	2729C>T	Ala910Val
	LdCL_330032400	ras-like small GTPase, putative	7A>C	Ser3Arg
	LdCL_330036100	hypothetical protein	304G>A	Asp102Asn
	LdCL_330036300	Concanavalin A-like lectin/glucanases superfamily/Beige/BEACH domain containing protein, putative	10099G>A	Gly3367Arg
	LdCL_330039900	Flagellar Member 8	7413G>T	Gln2471His
34	LdCL_340005500	DnaJ domain containing protein, putative	611C>T	Ala204Val
	LdCL_340006300	adenylate kinase, putative	228G>C	Glu76Asp
	LdCL_340007300	Antimony resistance marker of 58 kDa	188A>G	Tyr63Cys
	LdCL_340008100	calpain-like cysteine peptidase, putative	1189C>A	His397Asn
	LdCL_340008900	DNA-directed RNA polymerase III subunit C1, putative	4053G>T	Glu1351Asp
	LdCL_340015900	amastin-like protein	40T>C	Cys14Arg
	LdCL_340020500	hypothetical protein	1642C>G	Arg548Gly
	LdCL_340020700	hypothetical protein	5018G>A	Arg1673His
	LdCL_340027100	hypothetical protein	4148C>G	Ala1383Gly
	LdCL_340032200	phospholipid-transporting ATPase-like protein	4033G>T	Val1345Leu
	LdCL_340045300	hypothetical protein	2264C>A	Thr755Lys
	LdCL_340048800	hypothetical protein	4751C>T	Thr1584Ile
			6670G>A	Val2224Met
	LdCL_340050300	Unc104-like kinesin, putative	566C>A	Thr189Lys
35	LdCL_350009500	Arrestin (or S-antigen), N-terminal domain containing protein, putative	357G>T	Glu119Asp
	LdCL_350020500	hypothetical protein	2684C>T	Pro895Leu
	LdCL_350021000	hypothetical protein	530C>T	Ser177Leu
	LdCL_350023000	Eukaryotic rRNA processing protein EBP2, putative	506G>A	Arg169Gln
	LdCL_350030900	cytochrome P450 reductase, putative	531A>C	Glu177Asp
	LdCL_350045100	U5 snRNA-associated splicing factor	6981G>T	Lys2327Asn
	LdCL_350045500	mitochondrial carrier protein, putative	471G>C	Glu157Asp
	LdCL_350052200	hypothetical protein	329T>G	Leu110Arg
	LdCL_350055700	hypothetical protein	2212A>G	Thr738Ala
36	LdCL_360022900	tRNA pseudouridine synthase A, putative	2417G>C	Ser806Thr
	LdCL_360023700	hypothetical protein	2801C>G	Ala934Gly
	LdCL_360024400	MORN repeat, putative	1546C>T	Pro516Ser
	LdCL_360032100	hypothetical protein	353T>C	Leu118Ser
	LdCL_360034000	protein kinase, putative	10247C>T	Ala3416Val
	LdCL_360037300	NUC194 domain/Phosphatidylinositol 3- and 4-kinase, putative	4312G>A	Ala1438Thr
			3478T>C	Trp1160Arg
	LdCL_360039000	hypothetical protein	1777G>A	Val593Met
	LdCL_360050900	oxidoreductase, putative	749A>C	Lys250Thr
	LdCL_360051600	paraflagellar rod component, putative	2053A>G	Thr685Ala

Chrm	Gene ID	Product Description	SNP (gene)	SNP (protein)
	LdCL_360052300	trypanothione synthetase-like protein	1010C>G	Pro337Arg
	LdCL_360055600	hypothetical protein	686T>C	Val229Ala
	LdCL_360056400	Alpha/beta hydrolase family, putative	1835T>C	Phe612Ser
	LdCL_360056800	hypothetical protein	544C>T	Pro182Ser
	LdCL_360058500	hypothetical protein	4556A>G	Tyr1519Cys
			58G>A	Asp20Asn
	LdCL_360069200	kinetoplast DNA-associated protein, putative	479A>C	Lys160Thr
	LdCL_360071100	centrin, putative	133G>T	Asp45Tyr
	LdCL_360072300	flagellum transition zone component, putative	1087C>G	Leu363Val
	LdCL_360072400	ADP-ribosylation factor-like protein 3b, putative	166G>A	Val56Ile
	LdCL_360075200	protein of unknown function (DUF4496), putative	1060G>C	Asp354His
	LdCL_360080800	hypothetical protein	250A>G	Thr84Ala
	LdCL_360086100	acetyltransferase, putative	82A>G	Asn28Asp

*All SNPs from chromosome 1-36 along with the gene ID from TriTrypDB, gene name, genomic location of the SNP and protein location of the SNP are found in this table. SNPs relating to the ISC1 Yeti region were removed by intersecting with SNPs found in the samples with accession numbers ERR206282 and ERR206270.

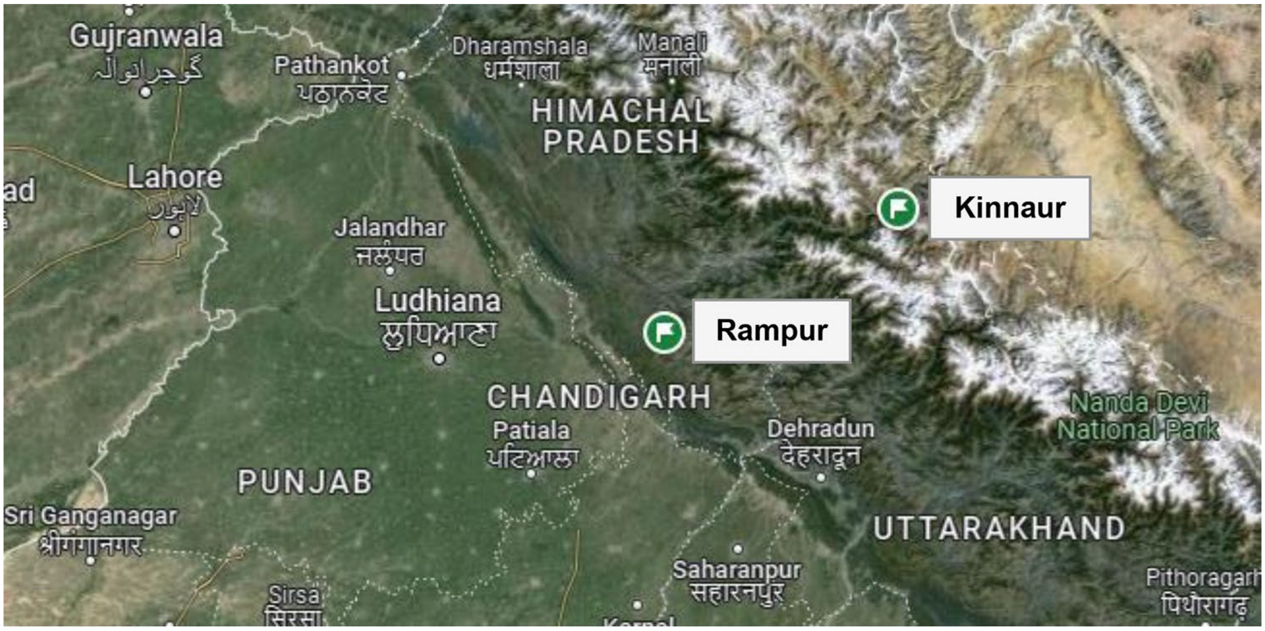

Appendix Figure 1. Location of patients presenting with LdHPCL71 and LdHPCL76. The Kinnaur (6050 m above sea level) and Rampur (1020 m above sea level) regions of Himachal Pradesh are indicated on this map and correspond to the location of isolation of LdHPCL71 and LdHPCL76 respectively. The map was generated using Google Maps.

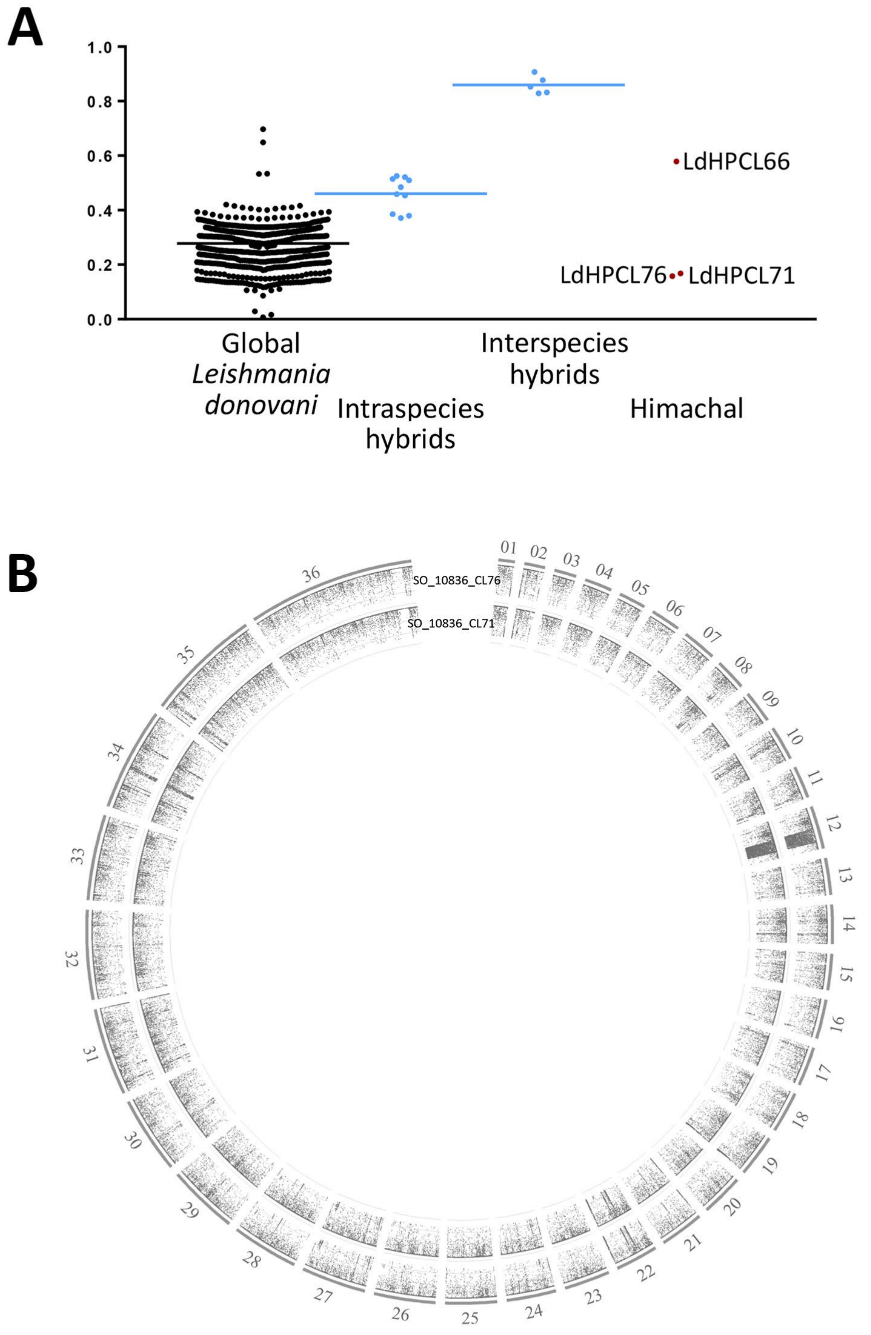

Appendix Figure 2. SNP heterozygosity of *L. donovani* isolated from Himachal Pradesh. (A) The heterozygous ratios of the new lineages (LdHPCL71, and LdHPCL76) from Himachal Pradesh are consistent with non-hybrid parasites, in contrast with the previously characterized LdHPCL66. (B) All SNP frequencies are plotted for LdHPCL76 (outer circle) and LdHPCL71 (inner circle) on their corresponding chromosome (1-36). A frequency = 1 is homozygous (outside of the circle), a frequency = 0.5 is heterozygous (middle of the circle) and a frequency = 0 means there is no SNP (inside of the circle). SNP analysis was performed against the SLCL *L. donovani* reference strain.
